# Nighttime Blue Light Promotes Lipid Accumulation and Shortens Lifespan via DAF-16/SOD-3-Mediated Oxidative Stress in *C. elegans*

**DOI:** 10.3390/genes17101263

**Published:** 2026-10-11

**Authors:** Zilong Cheng, Bayindala Xiagedeer, Qianqi Tu, Jian Zhang, Yinhan Li, Runhua Hao, Zhiyu Zhang, Yajiao Wu, Huan Tang, An Zhu, Hong Hu

**Affiliations:** 1 Department of Preventive Medicine, School of Public Health, Fujian Medical University, Fuzhou 350108, China; chengzi999410@163.com (Z.C.); m156336583@163.com (Q.T.); zhangjian0330@126.com (J.Z.); lyh960608@163.com (Y.L.); eros0423@163.com (R.H.); 18259155379@163.com (Z.Z.); tanghuan____6@163.com (H.T.); 2 Key Laboratory of Environment and Health, School of Public Health, Fujian Medical University, Fuzhou 350108, China; 3 Department of Toxicology, School of Public Health, Xinjiang Medical University, Urumqi 830017, China; bydlxagder@126.com; 4 Key Laboratory of Ministry of Education for Gastrointestinal Cancer, School of Basic Medical Sciences, Fujian Medical University, Fuzhou 350108, China; wuyajiao@fjmu.edu.cn

**Keywords:** blue light, shortens lifespan, lipid accumulation, oxidative damage, DAF-16/SOD-3

## Abstract

Background: Light, a crucial abiotic factor, consistently modulates organismal physiological activities. However, artificial light at night (ALAN) presents a considerable challenge. The widespread use of blue-light-rich light-emitting diodes (LEDs) in electronic products contributes to “blue light enrichment”. With population aging, the prevalence of obesity among older adults was expected to continue to increase. ALAN potentially induced obesity and shortened lifespan. However, the underlying pathogenic mechanism remains poorly understood. Methods: In this study, we investigated the effects of blue light on aging and lipid accumulation. Transcriptome and proteome analyses were performed to explore the potential regulatory mechanisms. Based on the enrichment results, we measured markers of lipid peroxidation and oxidative stress in *Caenorhabditis elegans* (*C. elegans*). We also conducted gene knockout experiments to verify the core regulatory genes. Results: The results showed that nighttime blue light induced lipid accumulation and aging in animals. Transcriptome and proteome analyses revealed that these effects might involve lipid transport, fatty acid metabolism, and oxidoreductase activity. Based on the enrichment results, we measured markers of lipid peroxidation and oxidative stress in *C. elegans*. Nighttime blue light exposure increased ROS, lipofuscin, lipid levels, and DAF-16 nuclear translocation, and decreased *daf-16*/*sod-3*. Notably, *sod-3* knockout further exacerbated nighttime blue-light-induced lipid accumulation and lifespan shortening. Conclusions: In conclusion, our observations in *C. elegans* suggest that blue light is associated with altered DAF-16/SOD-3 signaling and that SOD-3 appears to contribute to protection against blue-light-induced stress. These findings provide experimental evidence from *C. elegans* supporting minimizing unnecessary nighttime lighting, particularly blue light exposure.

## 1. Introduction

Light, an important abiotic factor in nature, is involved in the regulation of various metabolic pathways in human physiological activities [1]. The extensive use of artificial light at night (ALAN) considerably enhances human activity at night [2]; however, its potential negative effects on health require a comprehensive evaluation [3]. The brightness of the night sky increases by an average of 9.6% each year [4]. This illumination affects nighttime brightness and presents an increasing threat to human health [5]. Currently, more than 80% of the world’s population is affected by various forms of light pollution [6]. The widespread use of light-emitting diodes (LEDs), which emit substantial amounts of blue light, along with the growing use of electronic products, has made “blue light enrichment” increasingly common in the population [4,7].

Obesity is a growing and serious public health challenge, as well as a major risk factor for various chronic diseases, including cardiovascular diseases [8], metabolic disorders, cognitive impairment, and musculoskeletal disorders, which can lead to functional impairment and increased mortality rates [9]. Existing studies have demonstrated that nighttime light pollution, particularly nocturnal blue light exposure, is closely associated with human lipid metabolism disorders and obesity-related metabolic diseases [10]. Additionally, obesity affects health by increasing the strain on the healthcare system owing to disabilities and hospitalizations, particularly among older adults [11]. This challenge is intensified by the global shift toward an aging society. The World Health Organization (WHO) estimates that by 2050, the proportion of the global population aged over 60 years will reach 22% [12]. As the population ages, obesity among older adults is expected to become increasingly common. Among people over 65, the prevalence of obesity was approximately 40% [13]. The obesity epidemic, which is emerging in the shortened lifespan population, presents considerable public health problems.

The US National Toxicology Program identified ALAN exposure as an endocrine disruptor [14]. Epidemiological studies have shown that ALAN is a notably high risk factor for type 2 diabetes mellitus [15], cardiovascular disease, and obesity [16,17,18]. Blue light exerts significant biological effects across evolutionarily diverse model systems with conserved phototransduction. Long-term blue light exposure impairs the retina [19] and disturbs the metabolic regulation independent of visual perception in mammalian systems [20]. Blue light exposure results in a shortened lifespan and neurodegeneration in mice and *Drosophila* [21,22,23,24]. Currently, there is no direct evidence demonstrating that nighttime blue light exposure accelerates aging in human populations. Nevertheless, existing in vitro studies have shown that blue light exposure can promote senescence in human epidermal cells [25]. However, comprehensive studies investigating the physiological mechanisms underlying the effects of light wavelength on lipid metabolism remain limited. This study aimed to examine the effects of low-intensity nighttime blue light on lipid accumulation and lifespan reduction and explore the potential role of the SOD-3-mediated oxidative stress pathway in these processes. Currently, the International Commission on Illumination’s regulations regarding “blue light hazards” do not apply to non-visual effects. Considering the non-visual effects of blue light on human health, this study provides theoretical insight into the roles of blue light in aging and obesity.

## 2. Materials and Methods

### 2.1. Strain Preparation

Wild-type Bristol N2 *Caenorhabditis elegans*, specifically strains TJ356 (zIs356 IV [daf-16p::daf-16a/b::GFP + rol-6 (su1006)] and VC433 (sod-3(gk235) II), were obtained from the *Caenorhabditis* Genetics Center (CGC, Minneapolis, MN, USA). They were cultured on nematode growth medium (NGM) plates seeded with *Escherichia coli* OP50 at 20 °C in an incubator, as previously described [26]. The fertilized hermaphrodite nematodes were flushed from the culture dishes into conical tubes and lysed using a bleach solution. Synchronized L1-stage *C. elegans* larvae were obtained following the established protocol described in reference [27].

### 2.2. Light Treatment of C. elegans

#### 2.2.1. Different Wavelength Visible Light Exposure of *C. elegans*

**Figure 1:**
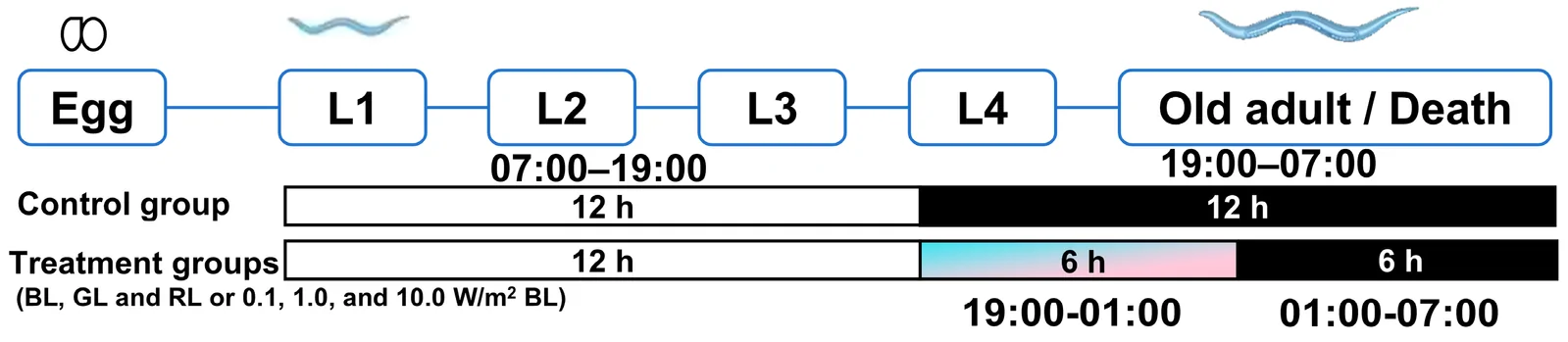
A schematic diagram of light treatment applied to *C. elegans*.

Experiments were conducted using LED light sources, and the spectra were measured [28]. *C. elegans* were synchronized to L1-larval and divided into four treatment groups: control, BL (blue light 450–480 nm), GL (green light 480–560 nm), and RL (red light 600–700 nm). All four groups were exposed to white light (300 lx) between 07:00 and 19:00. The three treatment groups were exposed to blue, green, and red light from 19:00 to 1:00 the subsequent day to simulate night light. When the white light illuminance was 300 lx, the corresponding irradiance was 0.9906 W/m^2^. To exclude the influence of light energy on the experiment, blue, green, and red light irradiances were set based on the irradiance of white light, with values of 1.00, 0.9205, and 0.9046 W/m^2^, respectively. The irradiance applied in this study was substantially below the threshold known to reduce *C. elegans’* lifespan [29]. After 3 days of exposure, *C. elegans* were collected for subsequent detection (Figure 1). A spectral irradiance colorimeter (Everfine, Hangzhou, China) was used to measure the illumination intensity and irradiance.

#### 2.2.2. Blue Light Exposure of *C. elegans*

L1-larval *C. elegans* were divided into four treatment groups: 0, 0.1, 1.0, and 10.0 W/m^2^ blue light (450–480 nm) treatment groups. All groups were exposed to white light (300 lx) from 07:00 to 19:00. Between 19:00 and 01:00, the three treatment groups were exposed to their respective irradiance of blue light. After 3 days, the *C. elegans* were collected for subsequent analysis. For the lifespan experiment, the three treatment groups were exposed to their respective blue light irradiances until all the *C. elegans* died (Figure 1).

### 2.3. Growth Assays

Following the different light exposures, *C. elegans* were observed under a SZX16 microscope (Olympus, Tokyo, Japan). Growth was assessed by measuring body length and width using CellSens Standard software (Olympus, Tokyo, Japan). Body length refers to the distance from the head to the tail in *C. elegans*, and body width measures the length of the widest part of the body of *C. elegans*. Twenty *C. elegans* were examined per treatment, and each experiment was repeated three times.

### 2.4. Locomotion Behavior Assay

The locomotor behavior of *C. elegans* is typically measured using head thrashing and body bending frequencies. The head thrash frequency was defined as the number of times the nematode head swung back and forth along the central axis of the body within 20 s. Body bend frequency was defined as the number of changes in the *C. elegans*’ posterior pharyngeal bulb along the *y*-axis in 1 min, assuming that *C. elegans* travels along the *x*-axis [30]. *C. elegans* treated with different light irradiation were transferred to a new plate without an OP50 NGM. Subsequently, 60 μL of M9 buffer (1 mM MgSO_4_, 5 mM KH_2_PO_4_, 20 mM Na_2_HPO_4_, and 20 mM NaCl) was added, and after adaptation for 1 min, the head thrash and body bend frequencies of *C. elegans* were calculated. Twenty *C. elegans* were examined per treatment, and each experiment was repeated three times.

### 2.5. Lipofuscin Levels

Following the different visible light exposure treatments, N2 and VC433 *C. elegans* were placed on 2% agar pads and anesthetized with sodium azide (10 nM) to observe intestinal autofluorescence. The intestinal autofluorescence detected in this study can largely reflect lipofuscin accumulation, which is a widely used surrogate marker of a shortened lifespan (Excitation = 365 nm; Emission = 420 nm). Images were obtained using an Axio Imager M2 microscope (Carl Zeiss Microscopy GmbH, Oberkochen, Germany), and lipofuscin levels were detected using ZEN 2 Pro software (Chinese Academy of Sciences, Beijing, China). Twenty *C. elegans* were included in each group, and three independent assays were performed.

### 2.6. Reactive Oxygen Species (ROS) Accumulation

To measure ROS accumulation in *C. elegans*, the oxidation-sensitive fluorescent probe 2′,7′-dichlorofluorescein diacetate (DCFH-DA) was used. L1-stage *C. elegans* exposed to different light treatments (BL, GL, RL, or 0.1, 1.0, and 10.0 W/m^2^ BL) for 72 h, as well as L4-stage N2 and VC433 strains, were incubated with 10 μM DCFH-DA probe for 120 min at 20 °C in the dark. After incubation, cells were washed twice with M9. *C. elegans* were placed on 2% agar pads and anesthetized with sodium azide. Images were obtained using an Axio Imager M2 microscope (Zeiss), and ROS accumulation was detected using the ZEN 2 pro software (Chinese Academy of Sciences) or Imagej software v1.54f (National Institutes of Health Bethesda, MD, USA). Twenty *C. elegans* were included in each group, and three independent assays were performed.

### 2.7. Total RNA Extraction and cDNA Library Construction

After different visible light exposure treatments (control and 1.0 W/m^2^ BL), 2000 synchronized L4-stage wild-type Bristol N2 *C. elegans* per group were lysed for total RNA extraction using TRIzol reagent (Invitrogen, Waltham, MA, USA). Briefly, the nematodes were collected in 1.5 mL centrifuge tubes with S-basal buffer, centrifuged at 3500 rpm for 5 min, and the supernatant was discarded; this washing step was repeated three times. An appropriate amount of TRIzol reagent was then added to the nematode pellet at a ratio of 1 mL TRIzol per 50–100 mg of nematodes. Grinding beads were added to the tubes, and the nematodes were fully lysed using a tissue grinder. The homogenate was collected and incubated at room temperature for approximately 5 min to completely dissociate nucleoprotein complexes. Subsequently, chloroform was added at a ratio of 0.2 mL per 1 mL of TRIzol; the tubes were tightly capped, vigorously shaken for about 15 s, and then incubated at room temperature for 2–3 min before centrifugation at 13,500 rpm for 15 min, during which the sample separated into three layers: a colorless upper aqueous phase containing RNA, a white interphase protein layer, and a red lower organic phase. The upper aqueous phase was carefully transferred to a new centrifuge tube, mixed with an equal volume of isopropanol by inversion, incubated at room temperature for 10 min, and then centrifuged at 12,000 rpm for 10 min to precipitate the RNA at the bottom of the tube. After discarding the supernatant, the RNA pellet was washed with 1 mL of 75% ethanol by centrifugation at 7500 rpm for 5 min; the ethanol was then discarded, and the wash step was repeated once more, with all residual liquid carefully removed. The centrifuge tubes were then air-dried in a laminar flow hood or under vacuum, and an appropriate amount of RNase-free water was added to gently resuspend the RNA pellet by pipetting, with dissolution facilitated by incubation in a 55–60 °C water bath for 10 min. Finally, total RNA was extracted and measured for concentration and integrity using the Nanodrop^TM^OneC spectrophotometer (Thermo Fisher Scientific Inc., Waltham, MA, USA) and 1.5% agarose gel electrophoresis, respectively. An A260/A280 ratio between 1.8 and 2.0 was considered acceptable.

Oligo (dT) magnetic beads were used to separate poly(A)-containing mRNA, and a fragmentation buffer was added to disrupt the mRNA into 300 bp short fragments. First-strand cDNA was synthesized using reverse transcriptase and random oligonucleotides, with fragmented mRNA as the template. Subsequently, the RNA strand was degraded by RNase H, and second-strand cDNA was synthesized from the dNTPs using DNA polymerase I. Double-stranded cDNAs were processed for end repair and adapter ligation. This was followed by PCR amplification and purification to obtain a cDNA library.

### 2.8. Quantitative Real-Time Polymerase Chain Reaction

The cDNA samples were diluted and analyzed using the SYBR Green Pro Taq HS Premix qPCR Kit II (AG11719, Accurate Biotechnology (Hunan) Co., Ltd., Changsha, China). The qPCR reaction mixture was prepared in a total volume of 20 μL. Quantitative real-time PCR (qPCR) was performed using an Agilent AriaMx Real-Time PCR System (Agilent Technologies, USA). The amplification conditions were as follows: initial denaturation at 95 °C for 30 s, followed by 40 cycles of denaturation at 95 °C for 5 s, and combined annealing/extension at 60 °C for 30 s. The data were processed using the 2^−ΔΔCt^ method for relative quantification. All experiments were performed with four biological replicates. The primer sequences used for qPCR are listed in Table 1.

### 2.9. DIA-Based Quantitative Proteomics Analysis

Data-independent acquisition (DIA)-based quantitative proteomics were employed to characterize the protein expression profiles of the samples in this study following exposure to blue light (control and 1.0 W/m^2^ BL) for 72 h starting at the L1 larval stage. Specifically, 25 mg of synchronized L4-stage nematodes was collected per group. Both the control group and the 1.0 W/m^2^ blue light exposure group were analyzed with three independent biological replicates to ensure reliable quantitative results. Total proteins were extracted from the samples via preprocessing, enzymatically digested into peptides with trypsin, and then subjected to mass spectrometric data acquisition in DIA mode using an Orbitrap high-resolution mass spectrometer. Retention time was calibrated with internal standard peptides to ensure the reproducibility of quantitative results. Raw data were processed through multiple preprocessing steps: proteins with missing values in more than 50% of the samples were filtered out, non-biological inter-sample biases were corrected via median normalization, and the remaining missing values were imputed using the minimum value imputation method. Differentially expressed proteins (DEPs) were screened by Student’s *t*-test with the thresholds of fold change (FC) ≥ 1.5 and *p* < 0.05. Functional annotation of DEPs was performed based on the COG, GO, and KEGG databases, followed by enrichment analysis using the hypergeometric distribution test. Subcellular localization of DEPs was predicted with the WoLF PSORT tool. Additionally, a protein–protein interaction (PPI) network was constructed based on the STRING database, with interaction pairs filtered by a combined score > 0.7 and hub core nodes further identified. All bioinformatic analyses were implemented using Python 3.9, R 4.3, and the BioLadder 2.0 platform. The proteomic mass spectrometry data have been deposited to the ProteomeXchange Consortium via the iProX repository with the dataset identifier PXD084495.

### 2.10. Transcriptome Sequencing and Bioinformatics Analysis

High-throughput mRNA-seq was performed by E-GENE Tech Co, Ltd. (Shenzhen, China). RNA and differential expression levels were analyzed using StringTie (Baltimore, MD, USA) [31] and DEseq2 [32]. Kyoto Encyclopedia of Genes and Genomes (KEGG) and Gene Ontology (GO) annotations were performed using DAVID (Frederick, MD, USA) [33]. Protein–protein interaction (PPI) analysis was performed using STRING software v11.0 (Swiss Institute of Bioinformatics, Zurich, Switzerland). The transcriptomic raw sequencing data have been submitted to the NCBI BioProject with BioProject ID: PRJNA1532578.

### 2.11. Lipid Staining

Oil Red O staining (Beyotime Biotechnology, Cat. No. C0157S, Shanghai, China) was performed to detect fat accumulation in *C. elegans* following exposure to blue light at different irradiances (control, 0.1, 1.0, and 10.0 W/m^2^). After 72 h of exposure starting at the L1 larval stage, N2 and VC433 *C. elegans* were dehydrated with 60% isopropanol, followed by incubation with Oil Red O staining solution for 60 min at 20 °C in the dark with shaking. Subsequently, the nematodes were washed three times with M9 buffer, transferred to a 2% agar pad, and imaged under an Axio Imager M2 microscope (Zeiss). Each treatment involved the examination of 20 nematodes, and each experiment was repeated three times.

### 2.12. Daf-16 Localization

Following exposure to blue light at different irradiances (BL, GL, RL, or 0.1, 1.0, and 10.0 W/m^2^ BL) for 72 h starting at the L1 larval stage, TJ356 *C. elegans* were collected, washed with M9 buffer, and anesthetized with levamisole. Nematodes were subsequently transferred to 2% agar plates for fluorescence imaging using an Axio Imager M2 (Zeiss) fluorescence microscope. Based on the distribution of green fluorescence, the nematodes were classified into cytoplasmic, intermediate, and nuclear groups. Each treatment involved the examination of 20 nematodes, and each experiment was repeated three times.

### 2.13. Determination of Superoxide Dismutase (SOD), Malondialdehyde (MDA), and Glutamic Oxaloacetic Transaminase (GOT)

#### 2.13.1. Determination of SOD Activity

*C. elegans* exposed to nighttime blue light for 3 days were collected (biochemical measurement was normalized to 1 mg *C. elegans* wet weight) in 1.5 mL centrifuge tubes with S-basal buffer. Samples were centrifuged at 3500 rpm for 5 min, the supernatant was discarded, and the washing procedure was repeated three times. The harvested nematodes were weighed and homogenized with normal saline at a ratio of 1:9 using grinding beads in a tissue grinder. The homogenate was transferred to a new tube and centrifuged at 4000 rpm for 5 min, and the supernatant was collected for subsequent assays. According to the protocols of the SOD assay kit (Nanjing Jiancheng Bioengineering Institute, Cat. No. A001-3-2, Nanjing, China), test and control samples were prepared and mixed thoroughly. The mixtures were incubated in a water bath at 37 °C for 40 min, followed by a 10 min incubation after adding the chromogenic reagent. The optical density (OD) was measured at 550 nm using a Thermo Scientific Multiskan GO microplate spectrophotometer (Thermo Fisher Scientific, Vantaa, Finland) to calculate SOD activity. All experiments were performed in three biological replicates.

#### 2.13.2. Determination of MDA Content

After a 3-day nighttime blue light exposure, *C. elegans* were collected (biochemical measurement was normalized to 1 mg *C. elegans* wet weight) and washed three times with S-basal buffer via centrifugation at 3500 rpm for 5 min. After weighing, the samples were mixed with MDA extraction solution at a ratio of 1:9 and fully homogenized. The homogenate was centrifuged at 8000 rpm for 10 min at 4 °C, and the supernatant was retained. Test and control tubes were prepared according to the instructions for the MDA assay kit (Solarbio Science, Cat. No. BC6415, Beijing, China). After thorough mixing, the samples were heated in a water bath at 100 °C for 60 min and then centrifuged at 10,000 rpm for 10 min at room temperature. The OD values were detected at 532 nm and 600 nm to determine MDA content. Three biological replicates were conducted.

#### 2.13.3. Determination of GOT Content

*C. elegans* exposed to nighttime blue light for 3 days were collected (biochemical measurement was normalized to 1 mg *C. elegans* wet weight) in 1.5 mL tubes, centrifuged at 3500 rpm for 5 min, and rinsed three times with S-basal buffer. The weighed samples were added with normal saline at a 1:9 ratio and homogenized. The homogenate was centrifuged at 4000 rpm for 5 min, and the supernatant was collected. Assay solutions were prepared in line with the manufacturer’s guidelines of the GOT detection kit (Nanjing Jiancheng Bioengineering Institute, C010-2-1). After mixing, the samples were incubated at room temperature for 5 min. The OD values were measured at 505 nm to quantify GOT content. The assay was repeated with three biological replicates.

### 2.14. Lifespan Assay

After exposure to blue light at different irradiances (Ctrl, 0.1, 1.0, and 10.0 W/m^2^), forty L4-stage nematodes from each group were transferred to fresh corresponding NGM plates. To inhibit egg laying, 50 μM of FUDR was added to the NGM medium, and 200 μL of *Escherichia coli* OP50 bacterial suspension was supplemented as the food source. The lifespan assay was performed with three independent biological replicates. *C. elegans* were considered dead when they failed to respond to gentle touch with a platinum wire pick. The day of *C. elegans* transfer and treatment initiation was defined as day 0 of the lifespan assay. All plates were incubated at 20 °C. The number of dead nematodes was recorded daily, and surviving worms were transferred to new NGM plates regularly until all individuals died. Finally, the survival curve was plotted based on the statistical data.

### 2.15. Lipid Peroxidation Measurement by BODIPY 581/591 C11 Probe

Intracellular lipid peroxidation was quantified using the BODIPY 581/591 C11 lipid peroxidation assay kit (Cat. No. S0043S, Beyotime Biotechnology, Shanghai, China) according to the manufacturer’s protocol. Briefly, treated L4-stage *C. elegans* were harvested and incubated with BODIPY 581/591 C11 working solution at a final concentration of 2 μM for 30 min at 20 °C under dark conditions. After washing off redundant unbound probe, fluorescence images were acquired immediately with an Axio Imager M2 microscope (Zeiss). Red fluorescence (non-oxidized probe): excitation = 581 nm, emission = 591 nm; Green fluorescence (oxidized probe): excitation = 488 nm, emission = 510 nm. Relative lipid peroxidation was calculated as the ratio of green fluorescence intensity to red fluorescence intensity.

### 2.16. Statistical Analysis

Measurement data were expressed as mean ± standard deviation, and SPSS 26.0 was used for statistical analysis. The experimental data were subjected to a one-way analysis of variance (ANOVA), and post-hoc pairwise comparisons were then performed using the Tukey HSD method. Differences between the two groups were assessed using an independent sample *t*-test, Survival lifespan data were analyzed via the Kaplan–Meier approach, and intergroup differences among survival curves were statistically compared using the log-rank test. Differences were considered statistically significant at *p* < 0.05. 

## 3. Results

### 3.1. Effects of Blue Light Exposure at Night on the Lipid Levels of C. elegans

**Figure 2:**
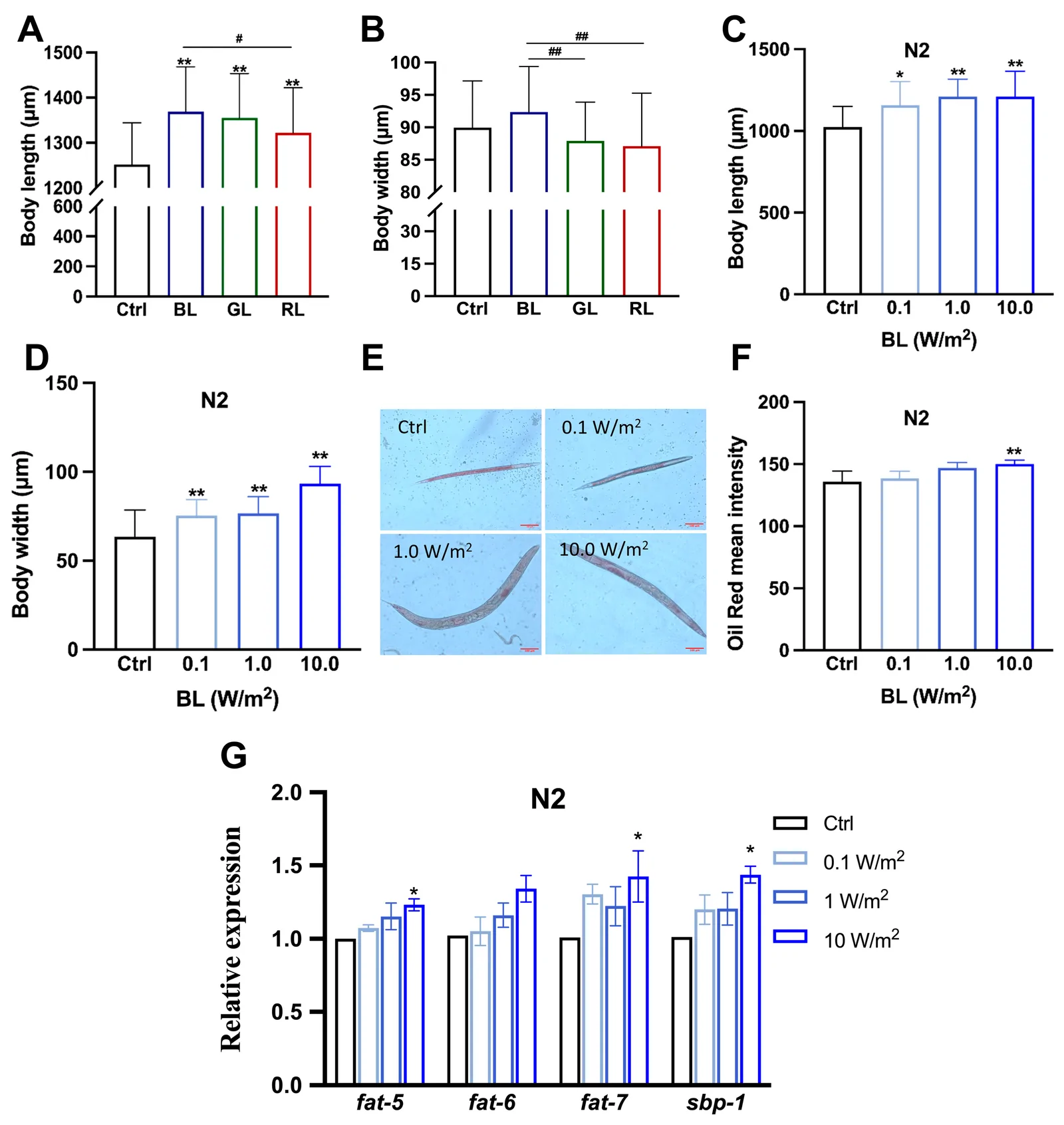
Effects of blue light exposure at night on the lipid levels of *C. elegans*. Body length (**A**) and body width (**B**) exposed to visible light at different wavelengths. Body length (**C**), body width (**D**), Oil Red O staining of N2 (**E**), and quantitative analysis (**F**) exposed to blue light. Expression of lipid biosynthesis and metabolic regulatory genes in N2 (**G**). (* *p* < 0.05 and ** *p* < 0.01, compared with the control group; # *p* < 0.05 and ## *p* < 0.01, compared among each treatment group).

We assessed the growth of *C. elegans* exposed to different nighttime visible light treatments. Growth was influenced by visible light wavelength. Compared with the control group, the body lengths of the treatment groups increased, with the BL group being longer than the RL group (Figure 2A). The body width increased in the BL group compared to the GL and RL groups (Figure 2B). These results indicate that blue light caused longer and wider *C. elegans* compared to those exposed to green and red light. With increasing blue-light dosages, the length, width, and lipid content also increased (Figure 2C–F). Lipid biosynthesis and metabolic regulatory genes (*fat-5*, *fat-6*, *fat-7*, and *sbp-1*) were significantly upregulated in the blue light treatment groups (Figure 2G).

### 3.2. Nighttime Blue Light Exposure Shortens the Lifespan of C. elegans

**Figure 3:**
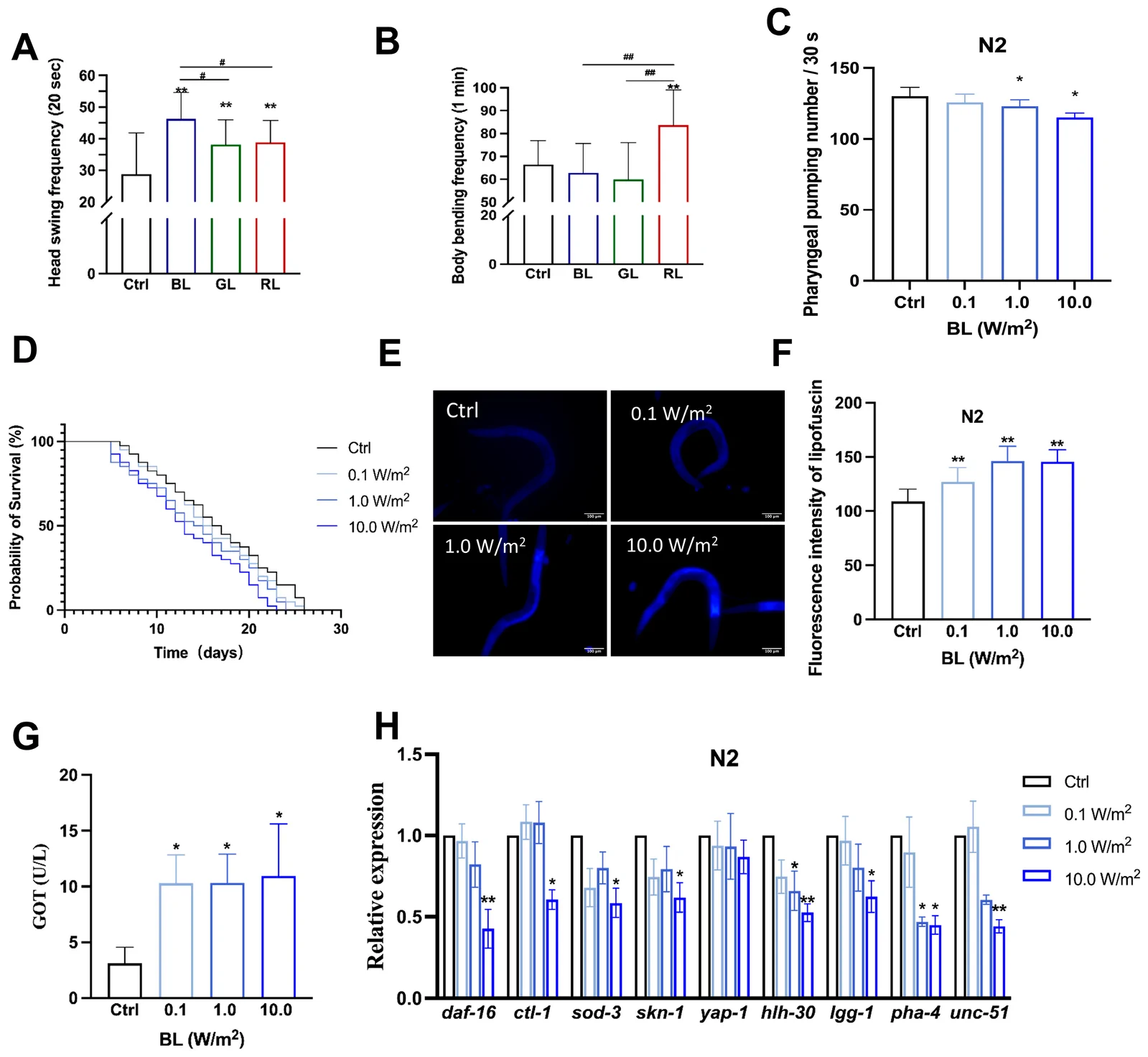
Nighttime blue light exposure shortens the lifespan of *C. elegans*. Visible light at different induced wavelengths increases head-swinging (**A**) and body-bending (**B**) frequency. Pharyngeal pumping frequency of *C. elegans* within 30 s (**C**). The lifespan of N2 exposed to blue light (**D**). The fluorescence intensity of lipofuscin in N2 (**E**,**F**). The GOT concentration of N2 (**G**). Expression of oxidative stress and lifespan-related genes in N2 (**H**). (* *p* < 0.05, and ** *p* < 0.01, compared with the control group; # *p* < 0.05, and ## *p* < 0.01, compared among each treatment group).

The frequency of head swinging and body bending was used to evaluate the locomotor behavior of *C. elegans*. The head swing frequency of the treatment groups increased compared to that of the control group. The head swing frequency in the BL group was higher than that in the GL and RL groups (Figure 3A). Compared to the control group, the frequencies of body bends in the BL and GL groups were not significantly different but were increased in the RL group (Figure 3B). In addition, the frequency of body bending was greater in the RL group than that in the BL and GL groups (Figure 3B). The effects of blue light with different intensities on the pharyngeal pumping frequency of *C. elegans*, specifically 1.0 and 10.0 W/m^2^ blue light, significantly suppressed pharyngeal pumping behavior (Figure 3C). Compared to the control group, blue light exposure reduced the lifespan of *C. elegans*, and this effect became more pronounced as the exposure intensity increased. The median lifespan for each group was 16.5 days (Control), 15.5 days (0.1), 14.5 days (1.0), and 13.0 days (10.0), the global Log-rank (Mantel–Cox) test across four groups yielded χ^2^ = 9.082, *p* = 0.0282 (Figure 3D). The fluorescence quantification of lipofuscin in *C. elegans* showed that, compared to the control group, the lipofuscin content in the blue light treatment group increased (Figure 3E,F), and the GOT also increased (Figure 3G). A q-PCR analysis revealed that, compared to the control group, genes related to oxidative stress and lifespan, including oxidative stress response (*sod-3*, *ctl-1*, *skn-1*), autophagy-lysosomal degradation (*lgg-1*, *unc-51*, *hlh-30*), and longevity regulation (*daf-16*, *pha-4*, *yap-1*) decreased (Figure 3H). Consistent with the aging-related phenotypes observed in *C. elegans*, preliminary mouse behavioral tests also indicated the detrimental biological effects induced by nighttime blue-light exposure (Supplementary Figure S1. Detailed experimental procedures are provided in Supplementary Methods). In conclusion, our results indicate that nighttime blue light exposure can lead to a shortened lifespan and increased oxidative stress levels in *C. elegans*.

### 3.3. Genome-Wide Transcriptional Profiling of C. elegans Exposed to Blue Light at Night

**Figure 4:**
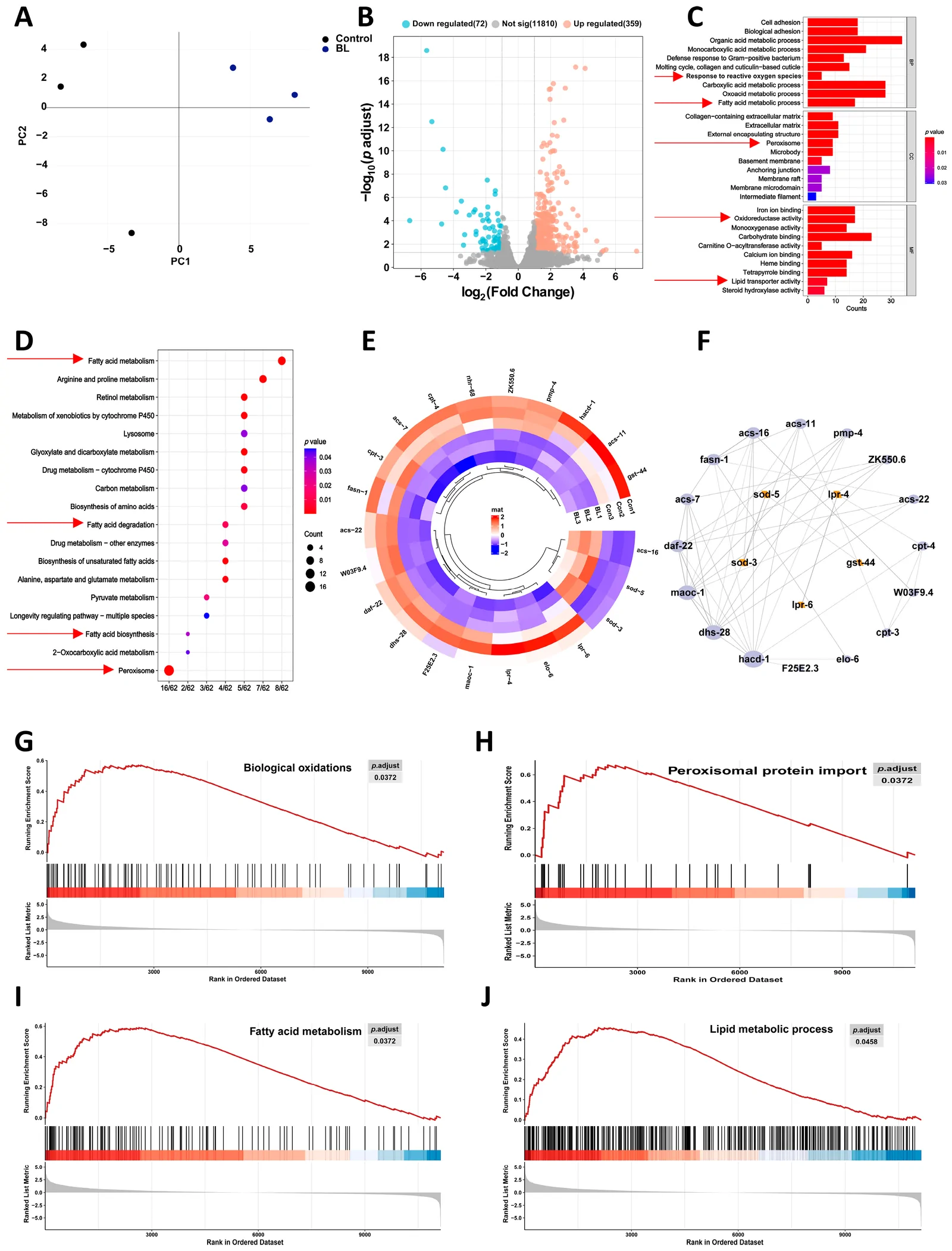
Genome-wide transcriptional profiling of *C. elegans* exposed to blue light at night. The PCA diagram of the control and 1.0 W/m^2^ BL groups (**A**). Volcano map of DEGs between the control and BL groups (**B**). The GO and (**D**) KEGG enrichment of DEGs (**C**). Heatmap of DEGs involved in both the fatty acid metabolic process and the response to reactive oxygen species (**E**). PPI analysis of the genes presented in the heatmap (**F**). GSEA analysis was conducted to analyze the effects of blue light exposure on REDOX homeostasis and lipid metabolism in nematodes (**G**–**J**).

To elucidate the mechanisms underlying the effects of light exposure on lipid accumulation in *C. elegans*, we performed an mRNA sequencing analysis. Following exposure to blue, green, and red light, only blue light tended to increase *C. elegans’* body width (Figure 2B). Therefore, blue light exposure was selected for subsequent transcriptome sequencing analyses. Principal component analysis (PCA) revealed significant intergroup differences and strong intragroup consistency between the control and BL groups (Figure 4A). We used stringent criteria (|log_2_ fold change| > 1 and adjusted *p* < 0.05) to detect differentially expressed genes (DEGs). After BL treatment, 431 DEGs were compared with those in the control group, of which 72 mRNAs were downregulated and 359 mRNAs were upregulated (Figure 4B).

GO enrichment analysis included subtypes of biological processes (BP), cellular components (CC), and molecular functions (MF). GO analysis of the DEGs indicated that BL mainly affected the fatty acid metabolic process, the response to reactive oxygen species, peroxisomes, oxidoreductase activity, and lipid transporter activity (Figure 4C). KEGG enrichment analysis indicated that BL primarily affected fatty acid metabolic processes, fatty acid degradation, and peroxisomes (Figure 4D). Changes in the expression of genes related to fatty acid metabolism and response to reactive oxygen species were observed (Figure 4E). We conducted PPI network analysis of these genes and arranged them according to their degree values. Genes associated with fatty acid metabolic processes are represented in purple, whereas those related to the response to reactive oxygen species are shown in orange. The results indicated that these genes were mutually regulated (Figure 4F). Notably, among the ROS-related genes, the *sod-3* gene had the most significant influence on the fatty acid metabolic process (Figure 4F). Gene set enrichment analysis (GSEA) revealed that biological oxidations, peroxisomal protein import, fatty acid metabolism, and lipid metabolism processes were activated (Figure 4G–J). These results indicate that blue light exposure primarily affects REDOX homeostasis and lipid metabolism in *C. elegans*.

### 3.4. Proteomic Analysis of C. elegans Exposed to Blue Light at Night

**Figure 5:**
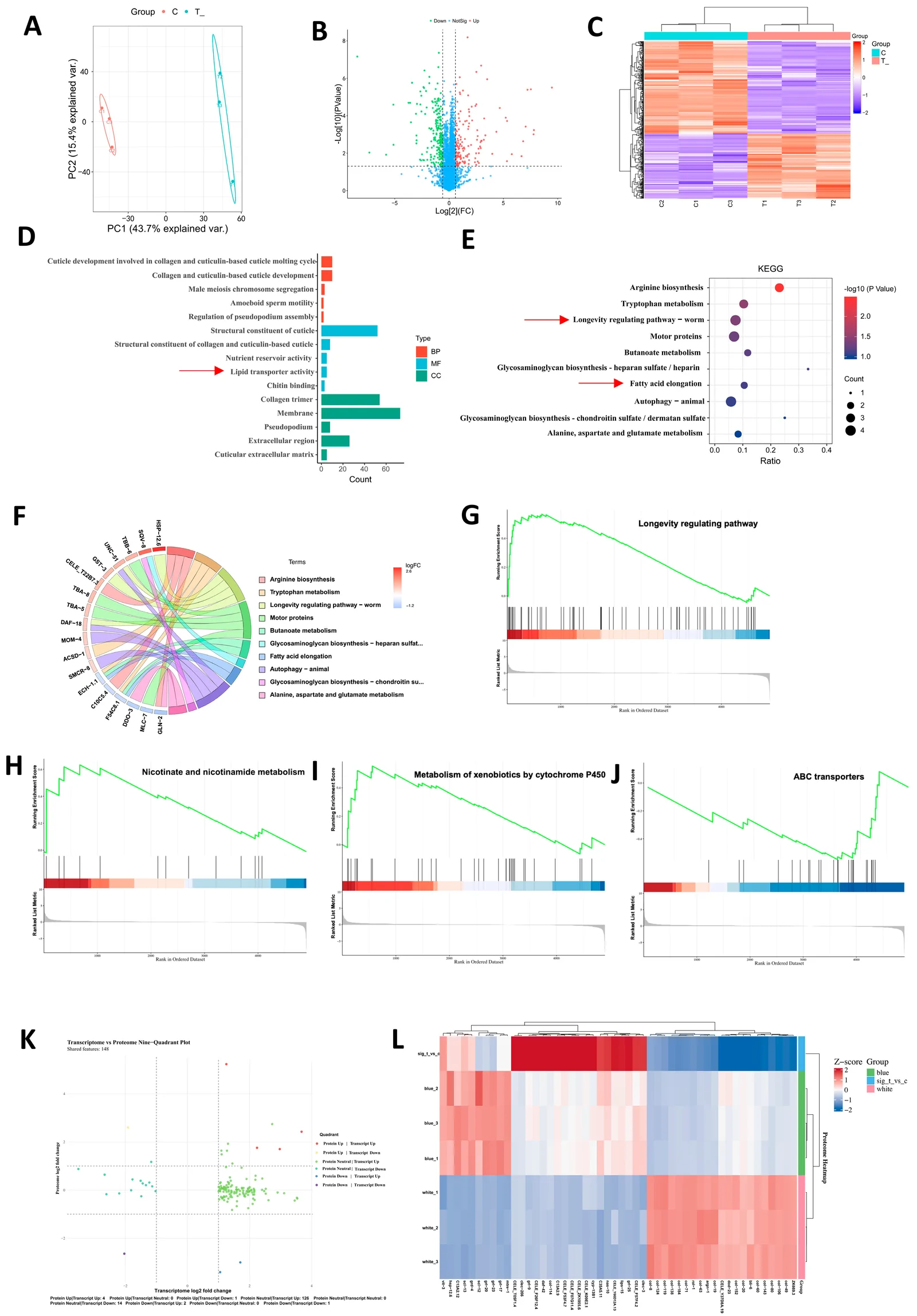
Proteomic Analysis of *C. elegans* Exposed to Blue Light at Night. The PCA diagram of the control and 1.0 W/m^2^ BL groups (**A**). Volcano map between the control and BL groups (**B**). Global clustering heatmap (**C**). The GO and (**D**) KEGG enrichment (**E**). Differentially Expressed Protein Chord Diagram (**F**). GSEA was performed to investigate the effects of blue light exposure on the longevity-regulating pathway, nicotinate and nicotinamide metabolism, metabolism of xenobiotics by cytochrome P450, and ABC transporters (**G**–**J**). Nine-Quadrant Diagram of Transcriptome-Proteome Association Analysis (**K**). Global Clustering Heatmap (**L**).

PCA revealed marked intergroup differences and strong intragroup consistency between the control group (Group C) and the blue-light-treated group (Group T) (Figure 5A). We used stringent criteria (|log_2_ fold change| > 1.5 and adjusted *p* value < 0.05) to identify differentially expressed proteins. In comparison with controls, a total of 382 differentially expressed proteins were detected following blue-light exposure, of which 224 proteins were downregulated and 158 proteins were upregulated (Figure 5B). The heatmap displays the functional classification and enrichment statistics for these differential molecules (Figure 5C).

GO analysis of the differentially expressed proteins showed that blue light exposure mainly altered lipid transporter activity (Figure 5D). KEGG enrichment analysis indicated that blue light affected the longevity-regulating pathway, fatty acid elongation (Figure 5E). These proteins were visualized in a differential protein chord diagram, where differentially expressed proteins are displayed on the left and sorted by logFC (blue for downregulation and red for upregulation). The right panel labels significantly enriched pathways, which intuitively reflects the global expression trends of each pathway in the treatment group (Figure 5F). GSEA revealed that the longevity-regulating pathway, nicotinate and nicotinamide metabolism, metabolism of xenobiotics by cytochrome P450, and ABC transporters were activated (Figure 5G–J).

Integrated transcriptome-proteome analysis was performed to explore blue light’s regulatory effects on *C. elegans*. A nine-quadrant diagram demonstrated a correlation between transcript and protein expression, with most showing consistent trends and some inconsistent, indicating post-transcriptional regulation (Figure 5K). A global clustering heatmap further displayed distinct protein expression patterns and significant changes in key functional pathways in the treatment group (Figure 5L).

These results indicated that nighttime blue light exposure mainly affects longevity regulation, lipid metabolism, and biological oxidations in *C. elegans*.

### 3.5. Effects of Blue Light Exposure at Night on Oxidative Damage in C. elegans

**Figure 6:**
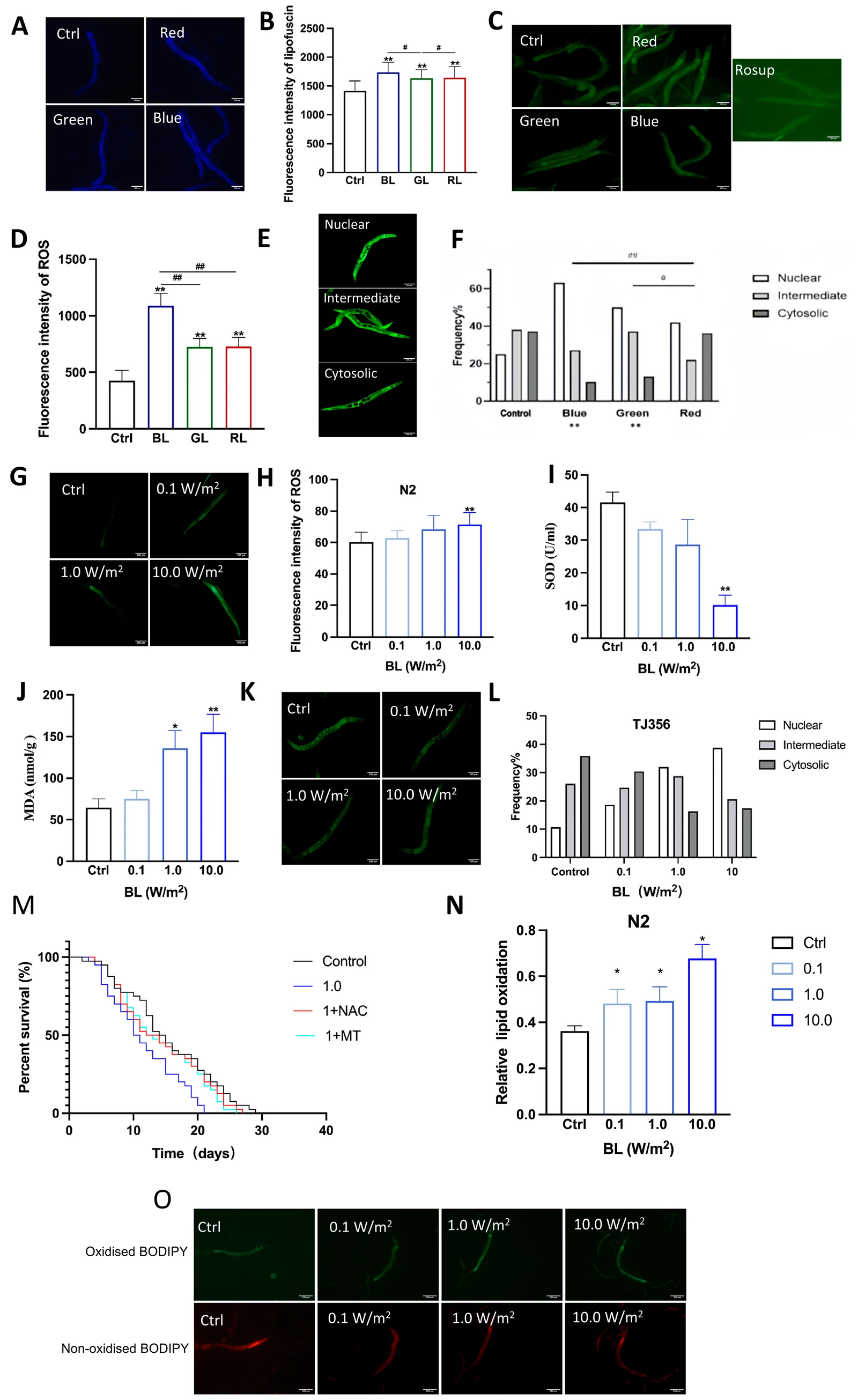
Effects of blue light exposure at night on oxidative damage in *C. elegans*. Representative images of intestinal lipofuscin in each group (**A**) and quantitative statistics of the relative fluorescence intensity of lipofuscin (**B**). Representative images of each group stained with the DCFH-DA probe (**C**) and quantitative statistics of the relative fluorescence intensity of ROS accumulation (**D**). Representative images showing the subcellular localization of *daf-16* in TJ356 (**E**). The GFP signals were classified as nuclear localization, transition state, or cytoplasmic localization (**F**). Representative images of the BL group stained with the DCFH-DA probe (**G**) and quantitative statistics of the relative fluorescence intensity of ROS accumulation (**H**). The SOD content of each group (**I**). The MDA content of each group (**J**). Representative images of the subcellular localization of *daf-16* in strain TJ356 (**K**) and its qualitative statistical results (**L**). The lifespan curves of the N2 nematode (**M**). Quantification of relative lipid oxidation levels was determined by calculating the ratio of green fluorescence intensity to red fluorescence intensity based on BODIPY-C11 staining (**N**) and representative images of each group stained with the BODIPY-C11 (**O**). The scale bar was 100 μm. * *p* < 0.05, and ** *p* < 0.01, compared with the control group. # *p* < 0.05; ## *p* < 0.01, compared between the two groups.

Lipids react with ROS to form lipid peroxide products, which cause oxidative stress and lead to the occurrence of oxidative damage. Secondary lysosomes in *C. elegans’* gut contain lipid peroxides that form lipofuscin. The nighttime blue-light-induced oxidative damage in *C. elegans* was verified by measuring intestinal lipofuscin levels, ROS accumulation, and DAF-16 localization. Intestinal lipofuscin levels were assessed by measuring the intestinal autofluorescence. The lipofuscin level and ROS of *C. elegans* in BL/GL/RL treatment groups increased compared to the control group (Figure 6A–D). Compared to GL and RL groups, a significant increase in ROS accumulation was observed in the BL group (Figure 6C,D). Compared to the control and RL (Red) group, the BL (Blue) and GL (Green) groups significantly enhanced DAF-16 nuclear translocation in *C. elegans* (Figure 6E,F). By quantifying the intestinal ROS fluorescence intensity in different blue-light-exposed groups, we observed that blue light exposure increased ROS accumulation (Figure 6G,H). By measuring the levels of SOD and MDA in *C. elegans*, blue light exposure decreased the SOD concentration (Figure 6I) in *C. elegans* while increasing the MDA level (Figure 6J). The treatment with 10 W/m^2^ blue light significantly enhanced DAF-16 nuclear translocation (Figure 6K,L). Supplementation with antioxidants (5 nM N-acetylcysteine, NAC; 100 mg/L melatonin, MT) significantly prolonged the lifespan of *C. elegans* compared to 1.0 W/m^2^ BL group. The median lifespan for each group was 14.5 days (Control), 10.5 days (1.0), 13.0 days (1 + NAC), and 12.5 days (1 + MT), the global Log-rank (Mantel–Cox) test across four groups yielded χ^2^ = 12.47, *p* = 0.0059 (Figure 6M). A BODIPY-C11 fluorescent probe assay was performed to comparatively analyze lipid peroxidation levels in *C. elegans*. The results confirmed that blue light exposure significantly increases lipid peroxidation (Figure 6N,O). These results indicated that nighttime blue light exposure causes oxidative damage and lipid accumulation in *C. elegans*.

### 3.6. Sod-3 Knockout Aggravated Lipid Accumulation and Shortened Lifespan Induced by Nighttime Blue Light

**Figure 7:**
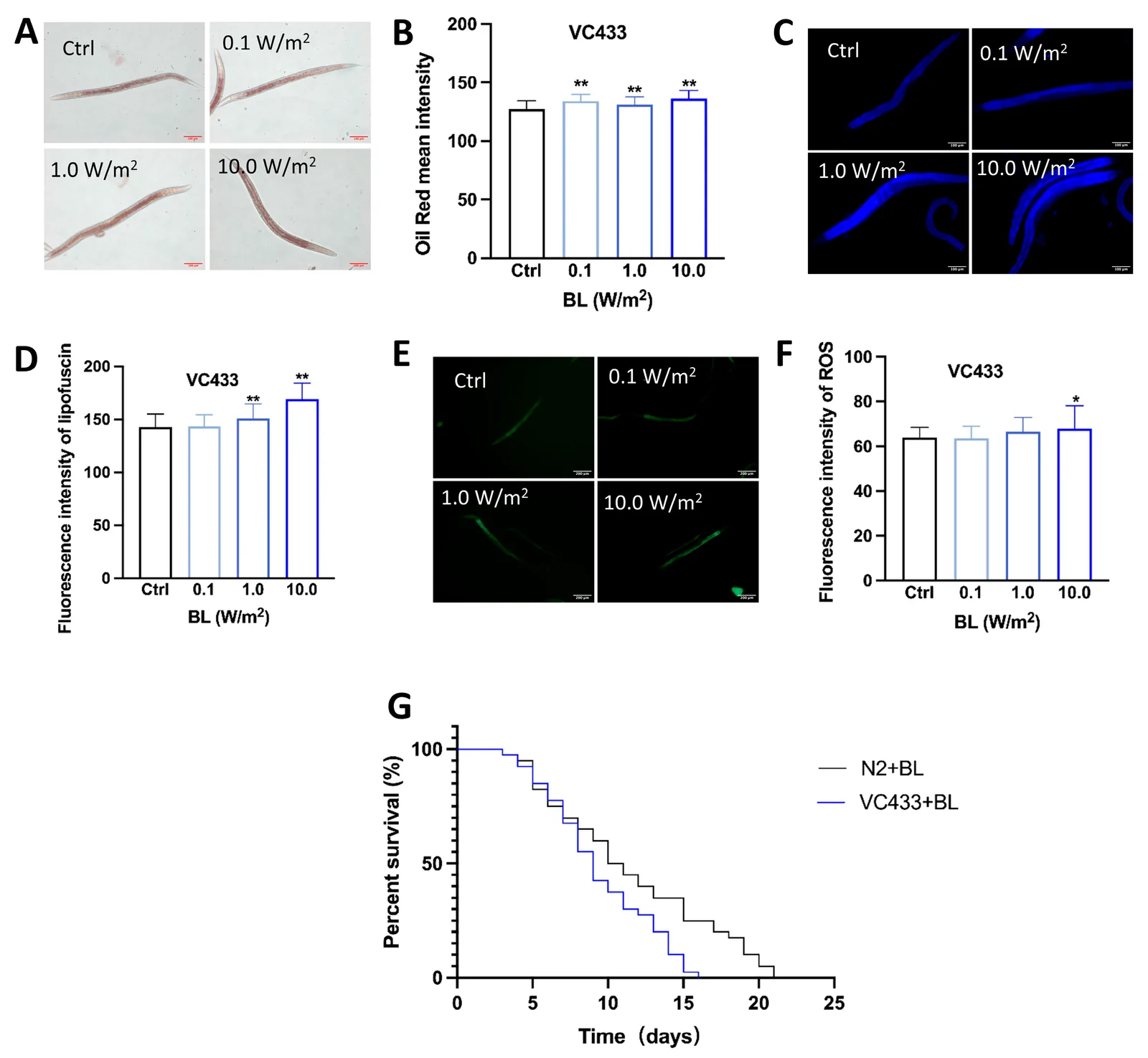
*Sod-3* knockout aggravated lipid accumulation and shortens lifespan induced by nighttime blue light. Representative images of Oil Red O staining of VC433 *C. elegans* (**A**) and quantitative analysis results of each group (**B**). Representative images of lipofuscin fluorescence in VC433 *C. elegans* (**C**) and the quantitative analysis results of each group (**D**). Representative images of ROS fluorescence in VC433 *C. elegans* (**E**) and the quantitative analysis results of each group (**F**), Survival curves of N2 and VC433 with BL exposure (**G**). * *p* < 0.05, and ** *p* < 0.01, compared with the control group. The scale bar is 100 μm (**A**,**C**), 200 μm (**E**).

PPI analysis indicated that the SOD-3 significantly influenced fatty acid metabolism, and qPCR showed that the expression of genes related to oxidative stress and lifespan such as *sod-3* was significantly downregulated after blue light exposure compared with the control group. Therefore, we used VC433 *C. elegans*, which has a *sod-3* gene knockout, to investigate the role of the *sod-3* gene in nighttime blue-light-induced lipid accumulation and shortened lifespan. The results of Oil Red O revealed that the knockout of the *sod-3* gene in VC433 *C. elegans* led to significant lipid accumulation following blue light exposure (Figure 7A,B) and exacerbated the accumulation of intestinal lipofuscin (Figure 7C,D) and ROS (Figure 7E,F). When exposed to blue light at 1.0 W/m^2^ for 6 h per day, VC433 had a shorter lifespan than wild-type N2. The median lifespan for each group was 9.0 days (N2 + BL) and 10.5 days (VC433 + BL), and the Log-rank test across two groups yielded X^2^ *=* 6.653, *p* = 0.0099 (Figure 7G). This indicates that loss of *sod-3* promotes blue-light-induced lipid accumulation and shortens lifespan.

## 4. Discussion

The American Medical Association issued a policy statement citing concerns regarding the health outcomes of ALAN [34,35]. ALAN has been identified as a potential risk factor for chronic diseases, particularly obesity [36,37,38]. Although the effects of ALAN on obesity have been extensively investigated, the mechanisms underlying its potential harmful impacts require further exploration. One hallmark of aging is the reduced energy efficiency in metabolic switching between carbohydrates and fatty acids, according to their relative availability [39]. Therefore, it is unclear whether ALAN contributes to obesity as well as a shortened lifespan. Phototransduction is conserved in worms and vertebrates [40]. *C. elegans* does not have a classical, mammalian-like circadian clock with the canonical feedback loop architecture (Cry). However, *C. elegans* does exhibit light- and temperature-entrainable physiological oscillations that represent a more ancestral form of circadian regulation [41], which affects gene expression, motor activity, and oxidative stress [42,43,44]. This renders *C. elegans* a suitable model for studying ALAN-induced health impairments. Therefore, this study aimed to investigate the effects of nighttime blue light on lipid metabolism and aging in *C. elegans*, as well as the potential underlying molecular mechanisms, to provide a preliminary theoretical basis for the effects of ALAN on human health.

Blue light exposure at night affected lipid accumulation and aging with a low dose. Our results demonstrated that nocturnal blue light significantly suppresses pharyngeal pumping in *C. elegans*, indicating that the food ingestion rate of nematodes does not increase. This finding ruled out overconsumption as the cause of increased body size and excessive lipid deposition, suggesting that the observed lipid accumulation arose from lipid metabolic dysfunction rather than elevated food intake. Blue light at night increased body width, reduced the lifespan, and altered the expression of lifespan-shortening genes at a dose of 1.0 W/m^2^. In contrast to other studies that investigated the effect of visible light on the lifespan of *C. elegans*, which was 16 W/m^2^ [29], the present study used a substantially lower level of blue light exposure. Although no population studies have shown that it can promote aging, our findings, combined with the alterations in the cognitive function of mice, proved that blue light induced universal aging in mammals and nematodes. The harmful effects of light pollution on glucose metabolism were synergistic with age and weight [45,46]. Compared with other parts of the light spectrum, nighttime light is more closely associated with blue light, which may contribute to the high prevalence of Type 2 diabetes and impaired glucose.

Lipid peroxidation and oxidative stress were core molecular events driving nighttime blue-light-induced lipid accumulation and lifespan shortening in *C. elegans*. Research indicated ALAN exposure disrupts metabolic rhythm, causing excessive caloric storage and subsequent weight gain [47]. Damage to retinal pigment epithelial cells by light was associated with oxidative stress [48]. Infrared light is low-energy long-wavelength light. Most existing studies have confirmed that infrared light exhibits extremely weak oxidative damage effects and does not induce significant lipid metabolic disorders [49]. Additionally, exposure to blue light of different intensities increased lipofuscin content and GOT levels, elevated MDA concentration, and reduced SOD activity. These results confirmed that blue light triggers lipid peroxidation (MDA, BODIPY-C11) and concomitant oxidative stress (ROS, SOD). Our previous study demonstrated that blue light reduces melatonin [28]. Melatonin, an endogenous free-radical scavenger, is sensitive to light in humans and mammals. Studies have shown that *C. elegans* also produces melatonin [50] and has a melatonin receptor [51]. Our study showed that melatonin and NAC, which are oxidative stress scavengers, ameliorated the adverse effects of blue light at night. These verified the oxidative stress induced by blue light exposure. Further, it explained how *C. elegans* may serve as a valuable model for studies on the oxidative stress caused by blue light.

The DAF-16/SOD-3 pathway appears to be a key regulatory node linking oxidative stress and fatty acid metabolic dysfunction. DAF-16 is located in the cytoplasm under physiological conditions, while under oxidative stress, *daf-16* translocates to the nucleus to activate the expression of antioxidant enzymes and plays an antioxidant role [52]. Blue light significantly decreased *daf-16* and enhanced the nuclear translocation of DAF-16. *Sod-3* encodes an iron/manganese superoxide dismutase, which can resist oxidative stress and promote a normal lifespan [53]. Blue light decreased *sod-3*, and transcriptomic PPI analysis identified SOD-3 as a key regulatory node linking oxidative stress and fatty acid metabolism dysfunction. The protein expression of *sod-3* was supported by proteomic evidence. As the antioxidant target of DAF-16, the significant downregulation of *sod-3* might be caused by decreased *daf-16*. Blue light may reduce the protective effect of SOD-3 by blocking the DAF-16-mediated antioxidant pathway, ultimately increasing oxidative stress damage. The *sod-3* knockout verified that SOD-3 functions in blue-light-induced lipid accumulation and shortens lifespan. The human FOXO family, homologous to *C. elegans* DAF-16, forms a conserved FOXO-SOD3 axis controlling antioxidant defense, lipid metabolism, and aging-related degeneration [54,55]. Nighttime blue light may disrupt this pathway, contributing to its potential harmful effects on human metabolic health and aging.

Nighttime blue light disrupted DAF-16/SOD-3 signaling, and changes in this pathway may contribute to blue-light-triggered lipid accumulation and a reduced lifespan. These findings provided a basis for future research into the potential health effects of blue-light-rich artificial light at night in humans. The limitations of the present study should be acknowledged. We have acknowledged that the lack of synchronized nematodes from identical batches prevents analysis of interaction between genotype and treatment for the *sod-3* mutant as a limitation of this study. Our investigation of DAF-16 regulatory patterns was restricted to its subcellular distribution and mRNA abundance of downstream antioxidant targets. We did not conduct biochemical assays to characterize DAF-16 post-translational modifications or quantify DAF-16 binding affinity to the *sod-3* promoter. Blue light may also alter the physiological state of OP50 bacteria, thereby indirectly influencing nematode phenotypes, and this potential interference cannot be fully excluded in the current study.

## 5. Conclusions

**Figure 8:**
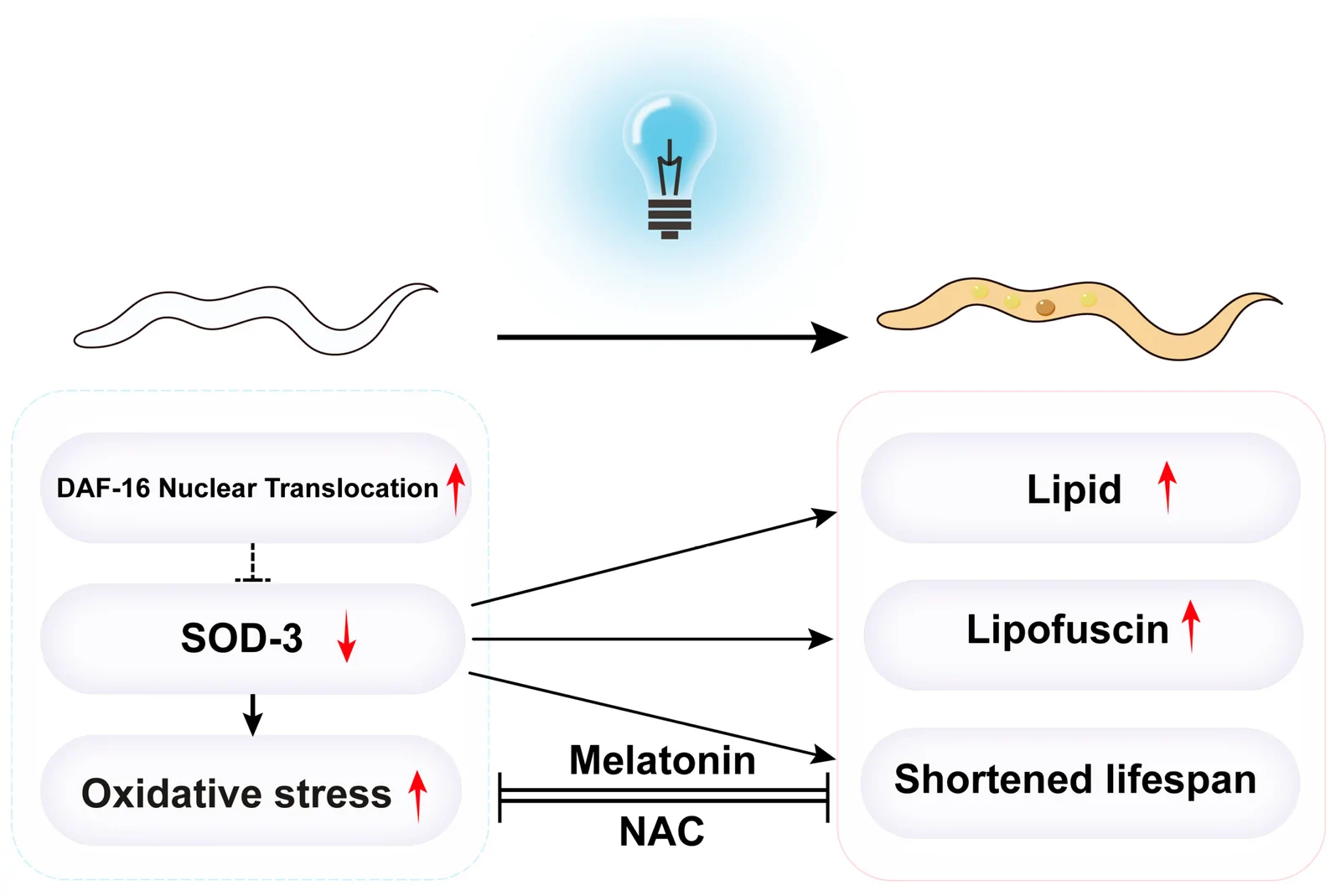
Schematic of the toxicity and mechanism of blue light at night on C. elegans.

Under our experimental conditions, nighttime blue light exhibits stronger detrimental effects on lipid metabolism and lifespan than green and red light in *C. elegans*. Nighttime exposure to blue light is associated with reduced DAF-16 abundance or impaired transcriptional function, which may lead to the downregulation of its downstream antioxidant effector SOD-3. This suppressed SOD-3 expression could, in turn, contribute to the elevated oxidative stress levels observed under blue light treatment. Our findings highlight the non-visual health risks of blue-light-rich artificial light at night and underscore the need to re-evaluate current nighttime blue light exposure standards (Figure 8). 

## Figures and Tables

**Table 1:** Primer sequences.

Gene	Primer Sequence (5′ → 3′)
*daf-16* (F)	TTCAAGCCAATGCCACTACC
*daf-16* (R)	TGCAGCTACAATTCCACACG
*ctl-1* (F)	CGGATACCGTACTCGTGATGAT
*ctl-1* (R)	CCAAACAGCCACCCAAATCA
*sod-3* (F)	ATGGACACTATTAAGCGCGA
*sod-3* (R)	GCCTTGAACCGCAATAGTG
*hlh-30* (R)	GCCGCTGCTCGTCCTATAAT
*yap-1* (F)	GCAGGACTCTCGGAAATACCAC
*yap-1* (R)	GCATGGGTAACTGCTCAACATC
*unc-51* (F)	AGTTCGGAGGATCGGAGAGT
*unc-51* (R)	TGGTATGCACTTGGACCTGC
*pha-4* (F)	AACCACGCAAGCACAGATGA
*pha-4* (R)	CCTGGATAACTGCTCGTTGTGT
*act-1* (F)	CAATGAGCTTCGTGTTGCCC
*act-1* (R)	AGGGAGAGGACAGCTTGGAT
*skn-1* (F)	TCAGACTCCACTGTCTCCTCT
*skn-1* (R)	AAACGAGTGTCTCTGTGAGTGA
*lgg-1* (F)	AACGCATCCAACTTCGTCCA
*lgg-1* (R)	TCGACCTCTCCTCCATACACA
*fat-5* (F)	GCCCTCTTCCGTTACTGCTT
*fat-5* (R)	CTCCGACTGCCGCAATAGAT
*fat-6* (F)	TCGCCGTGGATCCAAATGAA
*fat-6* (R)	ACGGTTTGCCATTTTGCCTC
*fat-7* (F)	TCGTTGCCATCACAAGTGGA
*fat-7* (R)	GAGTTTGCCTCCATGCTCCT
*sbp-1* (F)	CCGATCACCCGTCGAATCAT
*sbp-1* (R)	ACTTCCAGCGAGCAATCCAA
Using *act-1* as the internal reference	

## Data Availability

The raw transcriptomic sequencing data generated in this study have been deposited in the NCBI BioProject database: accession number: PRJNA1532578). The proteomic mass spectrometry data are available via ProteomeXchange with the dataset identifier PXD084495. All other data generated or analyzed during this study are available from the corresponding author on reasonable request.

## Supplementary Materials

Supplementary Materials: The following supporting information can be downloaded at: https://www.mdpi.com/article/10.3390/genes17101263/s1, Supplementary Methods; Figure S1: Blue light exposure alters the cognitive function of mice. The movement trajectory of mice in the novel object recognition experiment (**A**) and the novel object recognition index (**B**). Y-maze movement trajectory (**C**), total number of arm entries (**D**), and spontaneous alternation rate (**E**). Mouse nest building (**F**) and nesting score (**G**). (* *p* < 0.05, compared with the control group, n = 15).

## List of Abbreviations

ALAN	artificial light at night
BL	Blue light
CGC	Caenorhabditis Genetics Center
DCFH-DA	2′,7′-Dichlorodihydrofluorescein diacetate
DEG	Differentially expressed genes
DEP	Differentially expressed proteins
DIA	Data-independent acquisition
FUDR	5-fluoro-2′-deoxyuridine
GL	Green light
GO	Gene Ontology
GOT	Glutamic oxaloacetic transaminase
KEGG	Kyoto Encyclopedia of Genes and Genomes
LEDs	light-emitting diodes
MDA	Malondialdehyde
MT	melatonin
NAC	N-acetylcysteine
NGM	nematode growth medium
OD	Optical density
OP50	*Escherichia coli* OP50
PCA	principal component analysis
PPI	Protein–protein interaction
qPCR	Quantitative real-time PCR
RL	Red light
SOD	Superoxide dismutase
Tukey-HSD	Tukey’s honestly significant difference
WHO	World Health Organization
