# Supplementary material for: Nighttime Blue Light Promotes Lipid Accumulation and Shortens Lifespan via DAF-16/SOD-3-Mediated Oxidative Stress in C. elegans
Source: Genes (Basel). 2026 Oct 11;17(10):1263. doi: 10.3390/genes17101263 (PMC13704381; doi:10.3390/genes17101263)
Supplement: Supplementary file 1 [file genes-17-01263-s001.zip › Supplementary Figure S1.pdf]

Supplementary Figure S1

Using the novel object recognition test in mice, the novel object recognition index of mice decreased non-significantly under blue light exposure during the sleep period (BLS) than control group (Fig. S1 A-B). There was no significant difference in the total number of arm entries in the Y-maze among all groups, indicating that blue light exposure did not affect the overall locomotor activity of mice (Fig. S1 C-D). However, the spontaneous alternation rate, an indicator of working memory, was markedly lower in the BLS than in the control group Fig. S1 E). The nest-building score of the BLS group was lower than that of the control group (Fig. S1 F-G).

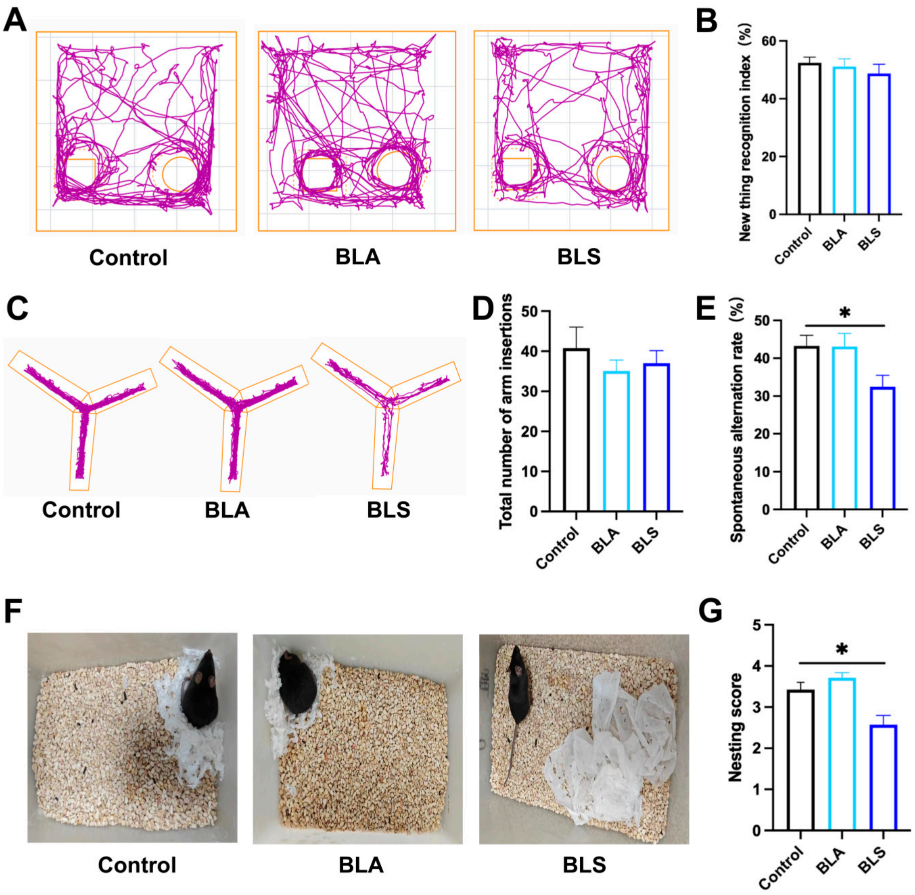

Supplementary Figure S1. Blue light exposure alters the cognitive function of mice. The movement trajectory of mice in the novel object recognition experiment (A) and the novel object recognition index (B). Y-maze movement trajectory (C), total number of arm entries (D), and spontaneous alternation rate (E). Mouse nest building (F) and nesting score (G). (**p* < 0.05, compared with the control group, n=15).
