# Supplementary material for: Nighttime Blue Light Promotes Lipid Accumulation and Shortens Lifespan via DAF-16/SOD-3-Mediated Oxidative Stress in C. elegans
Source: Genes (Basel). 2026 Oct 11;17(10):1263. doi: 10.3390/genes17101263 (PMC13704381; doi:10.3390/genes17101263)
Supplement: Supplementary file 1 [file genes-17-01263-s001.zip › Supplementary Methods.pdf]

Supplementary Methods

1.Measurement of mouse behavior

Forty-five C57BL/J mice were randomly divided into the control, blue light exposure during active period (BLA), and blue light exposure during sleep period (BLS) groups based on the body weight, with 15 mice per group. The experiment used LED light sources, and the spectrum was measured after a 5-day acclimation period. All three groups were maintained under white light (199.6 ± 1.2 lx, 6.11 ± 0.05 mW/cm²) on a 10 h light/14 h dark cycle from 10:00 to 20:00. The BLA group received additional light supplementation from 20:00 to 24:00 at 28.57 ± 0.91 lx (6.31 ± 0.18 mW/cm²), while the BLS group received supplementation from 06:00 to 10:00 at 28.9 ± 1.05 lx (6.32 ± 0.19 mW/cm²). The mice underwent 8 weeks of light modeling. The light/dark cycle (10 h light: 14 h dark, light phase 10:00-20:00) was chosen to align with the nocturnal activity pattern of C57BL/6J mice, ensuring that the active phase coincided with darkness. BLA group received blue light supplementation during the early active phase (20:00-24:00), while BLS received supplementation during the late sleep phase (06:00-10:00), allowing us to compare the effects of blue light exposure at different circadian phases. The blue light irradiance was maintained at a level comparable to that of the control group to eliminate the influence of different light energies. A spectral irradiance colorimeter (Everfine) was used to measure light intensity and irradiance. Illuminance during the dark phase was set to 0 lx. After 8 weeks, behavioral tests, including nest building, Y-maze, and novel object recognition, were conducted. The experiment was approved by the Institutional Animal Care and Use Committee of Fujian Medical University (IACUC FJMU2025-Y-0232).

Nest Building Test：The nest building test was used to assess general activity and cognitive function in mice. After modeling, mice were housed individually with corncob bedding and acclimated for 2 days. On the test day, the bedding was refreshed, and four pieces of filter paper (5 cm × 5 cm) were placed in each cage. After 24 h of continuous observation and photography, nest building performance was evaluated using a four-point blind scoring system: 1 point, intact paper scattered randomly with no biting marks; 2 points, intact paper accumulated at the cage corner without biting; 3 points, partially torn paper gathered at the corner with no integrated nest; 4 points, largely torn paper formed an incomplete nest structure. Statistical analysis was performed on the scores.

Y-Maze Test：The Y-maze test was performed to evaluate spatial working memory. Mice were acclimated to the experimental environment at 24±1 °C for 1 h in advance. The Y-maze (325 × 75 × 120 mm) consisted of three arms designated A, B and C. Each mouse was placed at the end of arm A facing the inner wall and allowed to explore freely for 8 min. Locomotor tracks and arm entries were recorded by a behavioral analysis system. The spontaneous alternation rate was calculated as: (number of correct alternations) / (total arm entries − 2) × 100%. The maze was wiped with 75% ethanol between trials to eliminate odor interference, and ambient conditions were kept stable throughout the test.

Novel Object Recognition Test (NOR)：Novel object recognition was applied to evaluate recognition memory based on the innate preference of rodents for novel objects. The test was carried out in an open field arena (50 × 50 × 40 cm). Firstly, mice were placed in the arena for 8 min to adapt to the environment. On the next day, two identical odorless and immovable objects (A and B) were placed 10 cm away from the side walls. Each mouse was released midway between the two objects, and the exploration time within 2–3 cm of each object was recorded for 5 min. One hour later, one familiar object was replaced with a novel object. The mouse was reintroduced to the arena for another 5 min, and exploration durations for novel and familiar objects were documented. The arena was disinfected with 75% ethanol after each trial. The novel object recognition index (NOI) was calculated as: (exploration time for novel object) / (total exploration time for novel and familiar objects) × 100%.
